# Assessment of Arsenic Exposure by Measurement of Urinary Speciated Inorganic Arsenic Metabolites in Workers in a Semiconductor Manufacturing Plant

**DOI:** 10.1186/2052-4374-25-21

**Published:** 2013-10-11

**Authors:** Kiwhan Byun, Yong Lim Won, Yang In Hwang, Dong-Hee Koh, Hosub Im, Eun-A Kim

**Affiliations:** 1 Occupational Safety and Health Research Institute, Korea Occupational Safety and Health Agency, Munemi-ro 478 Gusan-dong, Incheon-city 430-711, Republic of Korea; 2 Neodin Medical Institute, Seoul, South Korea

**Keywords:** Inorganic arsenic, Semiconductor, Metabolites

## Abstract

**Objectives:**

The purpose of this study was to evaluate the exposure to arsenic in preventive maintenance (PM) engineers in a semiconductor industry by detecting speciated inorganic arsenic metabolites in the urine.

**Methods:**

The exposed group included 8 PM engineers from the clean process area and 13 PM engineers from the ion implantation process area; the non-exposed group consisted of 14 office workers from another company who were not occupationally exposed to arsenic. A spot urine specimen was collected from each participant for the detection and measurement of speciated inorganic arsenic metabolites. Metabolites were separated by high performance liquid chromatography-inductively coupled plasma spectrometry-mass spectrometry.

**Results:**

Urinary arsenic metabolite concentrations were 1.73 g/L, 0.76 g/L, 3.45 g/L, 43.65 g/L, and 51.32 g/L for trivalent arsenic (As^3+^), pentavalent arsenic (As^5+^), monomethylarsonic acid (MMA), dimethylarsinic acid (DMA), and total inorganic arsenic metabolites (As^3+^ + As^5+^ + MMA + DMA), respectively, in clean process PM engineers. In ion implantation process PM engineers, the concentrations were 1.74 g/L, 0.39 g/L, 3.08 g/L, 23.17 g/L, 28.92 g/L for As^3+^, As^5+^, MMA, DMA, and total inorganic arsenic metabolites, respectively. Levels of urinary As^3+^, As^5+^, MMA, and total inorganic arsenic metabolites in clean process PM engineers were significantly higher than that in the non-exposed group. Urinary As^3+^ and As^5+^ levels in ion implantation process PM engineers were significantly higher than that in non-exposed group.

**Conclusion:**

Levels of urinary arsenic metabolites in PM engineers from the clean process and ion implantation process areas were higher than that in office workers. For a complete assessment of arsenic exposure in the semiconductor industry, further studies are needed.

## Introduction

The production of semiconductors continues to increase, as semiconductors are essential in the manufacturing of electronics. In Korea, the semiconductor industry has grown rapidly over the last decade, and semiconductors produced in Korea comprise a large share of the world market. Semiconductor manufacturing requires some toxic materials, which poses a risk of exposure to workers in the industry. The semiconductor production process can be divided into 2 sequential sub processes: wafer fabrication and chip assembly.

The wafer fabrication process is a multiple-step sequence of photolithographic and chemical processing steps including diffusion, photolithography, etching, ion implant, and metal deposition, during which electronic circuits are created on a wafer. During the diffusion process, dopants diffuse and an oxide film is formed on the wafer. In the photolithography process, the circuit pattern is formed. In the etching process, unnecessary parts of the pattern are removed. In the ion implantation process, impurities such as arsenic or phosphorus ions are implanted in order to impart conductivity to the wafer. Finally, in the metal deposition process, the parts of the circuits are connected electrically by depositing a conductive metal on the silicon wafer to create the integrated circuits [1]. Arsine is widely used in the wafer manufacturing process, especially during ion implantation, to impart conductivity to the wafer. Because of this, workers in the ion implantation process can be exposed to arsenic from arsine [2,3].

Before and after each step of wafer fabrication, there is another step called clean process where the wafers are cleaned using a solution of hydrofluoric acid, sulfuric acid, phosphoric acid, hydrogen peroxide, nitric acid, or isopropyl alcohol. During the clean process after ion implantation, arsenic ion can be dissolved in the cleaning solution. Therefore, workers of the clean process can also be exposed to arsenic.

Usually, workers in semiconductor manufacturing are categorized into operators and preventive maintenance (PM) engineers. The operators, who handle the wafers, are exposed to relatively low concentrations of chemicals. The PM Engineers are responsible for the processes and the equipment; hence, they frequently take apart the equipment and clean the inner parts. Thus, they are likely to be exposed to high concentrations of the various chemicals [4].

Arsenic is abundant on the earth, but exposure to arsenic has adverse health effects. Acute arsenic toxicity affects many organ systems including the gastrointestinal, dermal, nervous, hematopoietic, and cardiovascular systems. Long-term exposure to non-lethal doses of arsenic has been found to induce peripheral neuropathy, anemia, cardiac and peripheral vascular disease, anemia, hyperkeratosis, and hyperpigmentation of the skin [5]. Inorganic arsenic induces cancer of the skin, liver, and lung [6]. Arsenic compounds may be absorbed after ingestion, inhalation, or skin contact and are excreted in the urine.

In the blood, pentavalent arsenate (As^5+^) can be reduced to trivalent arsenite (As^3+^) in the blood, which is taken up mainly by the liver [7]. As^3+^ is metabolized to monomethylarsonic acid (MMA) and dimethylarsinic acid (DMA) by oxidative methylation and reduction. In humans, these metabolic processes do not go to completion, and inorganic arsenic along with mono- and di-methylated metabolites are excreted in the urine [8]. The measurement of urinary arsenic levels is generally accepted as the most reliable indicator of recent arsenic exposure [9].

Arsenic is naturally present in water, soil, and food, and seafood contains high levels of organoarsenic compounds such as arsenobetaine and arsenocholine that are less toxicity to humans. These organoarsenic compounds usually do not interfere with the metabolism of inorganic arsenic [10,11]. However, if total arsenic in the urine is measured, intakes of arsenobetaine and arsenocholine from seafood will interfere with the detection of occupational exposure to inorganic arsenic. To prevent interference from arsenic of marine origin, urinary arsenic speciation analysis, which can separately measure inorganic arsenic and its main urinary metabolites, is used [12,13].

There have been several studies on the exposure of workers to toxic factors in the semiconductor manufacturing industries in Korea [14], but real time assessment of arsenic exposure among PM engineers of the ion implantation process, and especially of the clean process, has not yet been reported. A few studies have assessed arsenic exposure using urinary speciated arsenic metabolites in workers in the ion implantation process in the United States [15], the United Kingdom, [16] and Taiwan [17]. However, these studies did not examine workers of the clean process. In Korea, exposure of workers in semiconductor manufacturing industries to arsenic has not previously been studied using the measurement of urinary speciated arsenic metabolites as an index of biological exposure. In this study, to examine exposure to arsenic among workers of the semiconductor manufacturing process, we measured urinary speciated arsenic metabolites in PM engineers of the ion implantation and clean process areas.

## Materials and methods

### Subjects

To evaluate the level of arsenic exposure relatively, we selected exposed group and non-exposed group. Participants were recruited from workers at a semiconductor manufacturing company. All of the PM engineers from the clean process and ion implantation process areas (n = 8 and n = 14, respectively), except night shift workers and off-duty workers, participated in this study. The non-exposed group consisted of 14 office workers from another company who were not occupationally exposed to arsenic. Duty of the PM engineers of clean process was checking the equipment of the clean process where the wafers are cleaned before and after each step of wafer fabrication such as photolithography process or ion implantation process. PM engineers of ion implantation process did the similar job on the ion implantation equipment. All participants were men. Preliminary explanation for this study was presented to all the participants. Only workers who signed informed consent documents participated in the study. The participants were interviewed and data on smoking status, consumption of seafood (fish, seaweed, shellfish, and crustaceans) during the previous 3 days, and job description were recorded. At the end of a working weekday morning, a spot urine specimen was collected from each participant.

### Urinary arsenic analysis using high performance liquid chromatography-inductively coupled plasma mass spectrometry (HPLC-ICP-MS)

A blank specimen, standard material for a calibration curve, and urine specimens for analysis (worker and quality control [QC] specimens, diluted 3:1 in 1% nitric acid) were placed in ICP-MS vials, after filtering through a cellulose acetate (0.2 m) filter. Filtered specimens were analyzed by HPLC-ICP-MS (ELAN DRC-II, Perkin Elmer, USA) (Table 1). We used G-EQUAS, the German External Quality Assessment Scheme, with the G-EQUAS 50 reference values, to standardize our analyses. We measured each specimen 7 times (same medium, same concentration) and calculated the standard deviation. We multiplied the standard deviation by 3 to determine the method detection limit (MDL). The MDLs were 0.148 g/L, 0.197 g/L, 0.137 g/L, and 0.054 g/L for As^3+^, As^5+^, MMA, and DMA, respectively. In the analysis of urinary speciated arsenic metabolites from the study participants, there were 5 cases with As^5+^ below 0.197 g/L, which is the MDL. In 6 cases where the metabolite was not detected (ND), a value of half the MDL was assigned.

**Table 1:** Operating conditions for the HPLC and ICP-DRC-MS

**High-performance liquid chromatography**	
Solvent A	6 mM Ammonium cabonate
Solvent	20 mM Ammonium phosphate, monobasic
Flow Rate	1.2 mL/min
Column	Anion Exchange, Hamilton PRP-X100, 4.6 mm I.d. × 150 mm, 5 μm
Guard column	2.3 × 30 mm PRP-X100TM
Autosampler flush solvent	5% Methanol
Sample injection volume	50 μL
**Inductively coupled plasma- dynamic Reaction Cell- mass**	
**spectrometry (ICP-DRC-MS)**	
RF Power	1400 W
Monitored Ion m/z	91 (^75^As^16^O) for DRC
Detector mode	Dual
Measurement uints	Cps
Autolens	On
Sample units	μg/L
Dwell time	250 ms
Cell gas	High purity O_2_ Gas
Cell gas flow rate	0.50 mL/min
RPQ	0.45

### Statistical analysis

Student’s t tests were performed to compare the concentrations of urinary speciated inorganic arsenic metabolites by smoking status and seafood intake. An ANOVA was conducted to compare the concentrations of urinary speciated inorganic arsenic metabolites among the 3 groups (PM engineers from the clean process area, PM engineers from the ion implantation process area, and office workers). Urinary speciated inorganic arsenic metabolites were transformed to a logarithmic scale. Multiple regression analysis was conducted with the groups (clean process, ion implantation process, office work) as independent variables with adjustment for the effects of smoking and recent seafood consumption, which are well-known confounders of urinary arsenic values from occupational exposure. A p value of <0.05 was considered significant. Statistical analyses were conducted using SAS 9.2 (SAS Institute Inc., Cary, NC, USA).

## Results

Among the 35 study participants, 75% of clean process PM engineers (6 of 8), 38.46% of ion implantation PM engineers (5 of 13), and 35.71% of the non-exposed group (5 of 14) were current smokers. Five clean process PM engineers had consumed seafood during last 3 days, and 2 clean process PM engineers and 6 office workers had ever had seafood. Neither smoking nor seafood consumption had a significant effect on the level of urinary speciated inorganic arsenic metabolites (As^3+^, As^5+^, MMA, and DMA). It shows no statistical significant differences on speciated urinary inorganic arsenic metabolites (As^3+^, As^5+^, MMA, and DMA) among each group (Table 2).

**Table 2:** The concentrations of urinary speciated inorganic arsenic metabolites by smoking status and seafood intake

		**As**^**3+**^	**As**^**5+**^	**MMA**	**DMA**	**As**^**3+**^**+ As**^**5+**^	**As**^**3+**^**+ As**^**5+**^**+MMA**	**As**^**3+**^**+ As**^**5+**^**+MMA + DMA**
**No smoking**	**AM**	1.67	0.58	2.96	33.72	2.25	5.21	38.93
**(N = 22)**	**GM**	1.33	0.37	2.62	26.92	1.80	4.51	32.48
	**GSD**	1.90	2.74	1.71	2.05	1.92	1.74	1.91
	**Med**	1.17	0.45	2.89	29.45	1.53	4.57	33.85
	**Min**	0.62	0.10	0.62	6.30	0.72	1.33	8.22
	**Max**	6.51	2.52	6.50	88.30	7.80	14.30	93.15
**Smoking**	**AM**	1.56	0.37	3.13	32.94	1.94	5.07	38.01
**(N = 13)**	**GM**	1.70	0.27	2.85	26.86	1.76	4.69	32.12
	**GSD**	1.62	2.38	1.59	2.00	1.64	1.52	1.88
	**Med**	1.70	0.35	2.59	29.62	2.21	4.67	36.93
	**Min**	0.55	0.10	1.27	10.27	0.65	2.70	13.56
	**Max**	2.49	0.94	5.37	69.39	2.83	7.94	74.07
**P-value**		0.75	0.40	0.63	0.99	0.92	0.83	0.96
**No seafood**	**AM**	1.82	0.41	3.23	29.23	2.23	5.46	34.69
**(N = 22)**	**GM**	1.50	0.29	2.93	23.98	1.87	4.89	29.41
	**GSD**	1.83	2.40	1.59	1.95	1.78	1.61	1.85
	**Med**	1.45	0.35	2.93	25.73	1.62	4.97	32.16
	**Min**	0.65	0.10	1.08	6.30	0.84	1.92	8.22
	**Max**	6.51	1.28	6.50	60.18	7.80	14.30	65.15
**Seafood**	**AM**	1.30	0.66	2.68	40.54	1.96	4.64	45.18
**(N = 13)**	**GM**	1.16	0.40	2.37	32.65	1.64	4.09	37.99
	**GSD**	1.67	2.96	1.74	2.08	1.87	1.71	1.91
	**Med**	1.14	0.50	2.48	39.31	1.94	4.07	40.65
	**Min**	0.55	0.10	0.62	8.88	0.65	1.33	10.96
	**Max**	2.62	2.52	5.37	88.30	5.14	9.53	93.15
**P-value**		0.20	0.33	0.23	0.21	0.52	0.31	0.25

As^3+^: trivalent arsenic, As^5+^: pentavalent arsenic, GM, Geometric mean; GSD, Geometric standard deviation; N, Number of subjects; MMA, Monomethylarsonic acid; DMA, Dimethylarsinic acid, P-value: probability by t-test.

The results of the analysis of urinary speciated arsenic metabolite values are shown in Table 3, which gives the geometric means (GM) of the concentrations of As^3+^, As^5+^, MMA, and DMA for each group. The geometric mean of As3^+^ of clean process PM, ion implantation PM and office workers were 1.73 μg/L, 1.74 μg/L, and 0.95 μg/L, respectively, which showed significant difference (p < 0.05). The geometric mean of As5^+^ for clean process PM, ion implantation PM and office workers were 0.76 μg/L, 0.39 μg/L, 0.18 μg/L, respectively, which also showed significant difference (p < 0.05). MMA values showed a similar trend, i.e., 3.45 μg/L, 3.08 μg/L and 2.310 μg/L for clean process PM, ion implantation PM and office workers, respectively (p < 0.05). As3^+^ and As5^+^ value, summed up to represent inorganic arsenic level, were 2.58 μg/L, 2.23 μg/L and 1.17 μg/L for clean process PM, ion implantation PM and office workers, respectively, which shows significant difference (p < 0.05). Summed value of inorganic arsenic and MMA were 6.11 μg/L, 5.34 μg/L and 3.37 μg/L for clean process PM, ion implantation PM and office workers, respectively (p < 0.05). On the contrary, DMA values were statistically insignificant (p 0.08). They were 43.65 μg/L, 23.17 μg/L and 23.43 μg/L for clean process PM, ion implantation PM and office workers, respectively. Total inorganic arsenic metabolites values, which is the summation of As^3+^, As^5+^, MMA and DMA, were 52.32 μg/L, 28.92 μg/L and 27.57 μg/L for clean process PM, ion implantation PM and office workers, respectively, which showed statistically insignificant value (p 0.06). The GM of urinary As^3+^, As^5+^, and MMA in the clean process PM engineers were the highest, followed by that in the ion implantation PM engineers and the non-exposed group. The GM of the sum of DMA and inorganic arsenic metabolites was the highest in clean process PM engineers, followed by that in the non-exposed group and ion implantation PM engineers.

**Table 3:** The concentrations of urinary speciated inorganic arsenic metabolites (in µg/L) by work group

		**As**^**3+**^	**As**^**5+**^	**MMA**	**DMA**	**As**^**3+**^**+ As**^**5+**^	**As**^**3+**^**+ As**^**5+**^**+MMA**	**As**^**3+**^**+ As**^**5+**^**+MMA + DMA**
**Clean**	**AM**	1.87	0.95	3.60	48.70	2.82	6.42	55.12
**(N = 8)**	**GM**	1.73	0.76	3.45	43.65	2.58	6.11	51.32
	**GSD**	1.54	1.99	1.38	1.72	1.57	1.42	1.52
	**Med**	2.03	0.68	3.40	48.84	2.61	6.71	54.55
	**Min**	0.83	0.34	1.94	14.07	1.17	3.11	23.60
	**Max**	2.63	2.52	5.37	88.30	5.14	9.53	93.15
**Ion implantation**	**AM**	1.88	0.48	3.26	27.23	2.36	5.62	32.85
**(N = 13)**	**GM**	1.74	0.39	3.08	23.17	2.23	5.34	28.92
	**GSD**	1.51	2.11	1.42	1.76	1.43	1.40	1.66
	**Med**	1.86	0.50	2.96	19.94	2.30	5.51	25.86
	**Min**	0.76	0.10	1.84	12.28	1.21	3.25	16.35
	**Max**	4.09	0.94	5.37	69.39	4.53	9.89	74.07
**Office**	**AM**	1.26	0.27	2.47	30.47	1.53	4.00	34.47
**(N = 14)**	**GM**	0.95	0.18	2.10	23.43	1.17	3.37	27.57
	**GSD**	1.86	2.36	1.83	2.22	1.82	1.74	2.08
	**Med**	0.74	0.10	2.27	28.35	1.07	3.27	31.70
	**Min**	0.55	0.10	0.62	6.30	0.65	1.33	8.22
	**Max**	6.51	1.28	6.50	60.18	7.80	14.30	65.15
**All**	**AM**	1.63	0.50	3.02	33.43	2.13	5.15	38.59
**(N = 35)**	**GM**	1.36	0.33	2.71	26.90	1.78	4.58	32.35
	**GSD**	1.79	2.60	1.65	2.01	1.80	1.65	1.88
	**Med**	1.14	0.41	2.88	29.62	1.70	4.67	36.93
	**Min**	0.55	0.10	0.62	6.30	0.65	1.33	8.22
	**Max**	6.51	2.52	6.50	88.30	7.80	14.30	93.15
**P-value**		<0.05	<0.05	<0.05	0.08	<0.05	<0.05	0.06

As^3+^: trivalent arsenic, As^5+^: pentavalent arsenic, GM, Geometric mean; GSD, Geometric standard deviation, N, Number of subjects; MMA, Monomethylarsonic acid; DMA, Dimethylarsinic acid, P-value: probability by ANOVA test.

The result of the multiple regression analysis revealed that the concentrations of As^3+^, As^5+^, MMA, the sum of inorganic arsenic (i.e., As^3+^ + As^5+^), the sum of inorganic arsenic and MMA, and the total inorganic arsenic metabolites were higher in clean process PM engineers than in non-exposed group. The concentrations of As^3+^, As^5+^, the sum of inorganic arsenic (As^3+^ + As^5+^), and the sum of inorganic arsenic and MMA were also higher in ion implantation PM engineers than in the non-exposed group (Table 4).

**Table 4:** Multiple regression analysis of inorganic arsenic metabolites by work group

		**Estimate**	**Standard error**	**P-value**	**R**^**2**^
**As**^3+^*	**Ion implantation**	0.53	0.21	0.02	
	**Clean**	0.66	0.23	0.01	
	**Office**	reference			0.31
**As**^5+^*	**Ion implantation**	0.90	0.32	0.01	
	**Clean**	1.38	0.36	<0.01	
	**Office**	reference			0.40
MMA*	**Ion implantation**	0.30	0.19	0.12	
	**Clean**	0.56	0.21	0.01	
	**Office**	reference			0.24
DMA*	**Ion implantation**	0.03	0.27	0.92	
	**Clean**	0.60	0.31	0.06	
	**Office**	reference			0.16
**As**^**3+**^**+ As**^5+*^	**Ion implantation**	0.61	0.20	0.01	
	**Clean**	0.82	0.23	<0.01	
	**Office**	reference			0.37
**As**^**3+**^**+ As**^**5+**^+MMA*	**Ion implantation**	0.40	0.18	0.03	
	**Clean**	0.64	0.20	<0.01	
	**Office**	reference			0.31
**As**^**3+**^**+ As**^**5+**^+MMA + DMA*	**Ion implantation**	0.08	0.25	0.75	
	**Clean**	0.60	0.28	0.04	
	**Office**	reference			0.18

As^3+^: trivalent arsenic, As^5+^: pentavalent arsenic, GM, Geometric mean; GSD, Geometric standard deviation; MMA, Monomethylarsonic acid; DMA, Dimethylarsinic acid, Total: sum of inorganic arsenic metabolites (As^3+^+ As^5+^+MMA + DMA), * adjusted for smoking status, seafood intake.

## Discussion

The workers in semiconductor manufacturing plants can be exposed to various toxic substances. Among them, the arsenic used in the ion implantation process as arsine gas is one of the most significant problems. In this study, multiple regression analysis revealed that As^3+^, As^5+^, the sum of inorganic arsenic (As^3+^+ As^5+^), and the sum of inorganic arsenic and MMA were higher in ion implantation PM engineers than in the non-exposed group.

Previous studies showed that PM engineers who handle the normal processes of the fabrication operation are exposed to arsenic levels that are substantially lower than the TLV of 10 μg/m^3^ if the operation is appropriately maintained by engineering controls such as exhaust ventilation, enclosure, and shielding of the operation equipment [4]. However, the equipment, which is sealed during normal operations, is opened and disassembled by the PM engineers during the maintenance process; hence, they can be exposed to residual arsenic that adheres to the inner surfaces of the equipment [2,18]. In a Taiwanese study that monitored arsenic exposure during the maintenance of an ion implanter, arsenic levels were very low in environmental samples, but were much higher in some wipe samples, used cleaning cloths, and gloves [2]. The results indicated that arsenic intake via ingestion, rather than through inhalation, might play a significant role in the elevation of urinary arsenic levels of workers performing maintenance on an ion implanter [2].

In another Taiwanese study that monitored arsenic exposure in ion implanter PM engineers, the levels of total urinary inorganic arsenic metabolites in PM engineers were 1.7 g/L, 1.4 µg/L, 6.2 µg/L, 20.2 µg/L, 29.5 µg/L for As^3,+^, As^5+^, MMA, DMA, and total urinary inorganic arsenic metabolites, respectively. Further, both the concentration of MMA and its percentage of the total urinary inorganic arsenic metabolites were significantly higher in PM engineers than in the non-exposed group [17].

In the study of Hu et al. (2006) that evaluated the effects of arsenic exposure among semiconductor workers, the mean urinary concentrations of As^3+^, As^5+^, MMA, DMA, and total arsenic metabolites (the sum of As^3+^, As^5+^, MMA and DMA) were 2.19 g/L, 0.82 g/L, 3.86 g/L, 44.33 g/L, and 51.21 g/L for exposed workers, respectively, and exposed workers had significantly higher concentrations of MMA, DMA, and total arsenic metabolites than non-exposed workers [19]. In the Taiwanese studies [17,19], the levels of urinary arsenic metabolites of the exposed groups were similar to those of clean process PM engineers in our study.

In a British study that monitored concentrations of urinary arsenic compounds, the mean urinary concentrations of As^3+^, As^5+^, MMA, DMA, and total arsenic metabolites (sum of As^3+^, As^5+^, MMA, and DMA) were 0.6 µg/L, 0.2 µg/L, 0.7 µg/L, 4.9 µg/L, and 6.4 µg/L, respectively, for exposed workers in the semiconductor manufacturing industry [16]. These levels were lower than that in our study.

In our study, the levels of urinary arsenic metabolites in clean process PM engineers were higher than those in ion implantation process PM engineers and the non-exposed group. In an experimental study conducted by Ungers et al. (1985), silicon wafers were found to emit inorganic arsenic following ion implantation. Data collected during this experiment demonstrated that arsenic is released over a 3.5-hour period following implantation and that the total amount of arsenic emitted may approach 6.0 g per 100 wafers processed within 4 hours after implantation [3]. However, there is a lack of research evaluating arsenic exposure for clean process PM engineers.

Urinary arsenic is determined in a secondary confirmative test in special medical examinations in Korea, with the limit being 220 g/L [20]. ACGIH recommends total inorganic arsenic metabolites should not exceed 35 g/L as the biological exposure limit (BEL) [9]. An upper limit for speciated arsenic metabolites in the urine has not been established thus far in Korea. Our results show that the levels of total inorganic metabolites in 88% of clean process PM engineers exceeded the BEL set by ACGIH; further, the levels exceeded the BEL in 43% of the non-exposed group and 39% of the ion implantation PM engineers. Several office workers of the non-exposed group had levels that exceeded the BEL, which can be attributed to non-occupational environmental factors or diet, especially seafood intake. The average seafood intake is much higher in Asian countries such as Korea, Japan, and Taiwan, than in Europe or the United States [21]. Likewise, the concentration of urinary arsenic in the Asian population is higher than in Western countries [22,23]. Because of the apparent correlation between seafood intake and arsenic exposure, the BEL should be applied in a conservative manner and may not be suitable to assess health risks. A study of the general population in Japan showed that the median value of urinary inorganic arsenic metabolites (the sum of As^3+^, As^5+^, MMA, and DMA) was 54 g/L, which exceeded the BEL [22]. It is notable that certain arsenic metabolites can be found even in the general population in the absence of occupational arsenic exposure. Similar research results in Korea revealed that the average concentration of urinary arsenic in the general population was 118 μg/g creatinine [24], which is comparable to the level of 149 μg/g creatinine in Japanese study [25]; both values are substantially higher than the average urinary arsenic concentration in the general population of the United States, which was 8.30 μg/L or 8.24 μg/g creatinine [26].

Shellfish are rich in DMA, which when ingested is not metabolized, but directly excreted in the urine [27]. Arsenosugars, which can be found in seaweed, mussels, shellfish, and oysters, is metabolized to DMA in the human body and can be excreted in the urine [28]. One study demonstrated increased total urinary inorganic arsenic metabolites, especially DMA, in a group of volunteers after consuming seaweed. Thus, urinary DMA does not represent occupational exposure to arsenic, but is a marker of seafood intake [29]. Hakala et al. (1995) performed a study in copper smelter workers to assess occupational arsenic exposure; they showed that urinary inorganic arsenic (As^3+^, As^5^) is more useful for assessing occupational exposure to arsenic, rather than total urinary inorganic arsenic metabolites [30]. In this study, the concentration of inorganic arsenic (As^3+^, As^5^) was significantly different between groups. Because seafood consumption in Asian countries is higher than in Western countries, Hata et al. (1995), on the basis of their findings in the general population in Japan, recommended excluding DMA when assessing occupational exposure to arsenic [22]. We analyzed the urinary inorganic arsenic metabolites As^3+^, As^5+^, and MMA (excluding DMA), and found a significant difference between the exposed and the non-exposed groups. The DMA concentration, however, was not significantly different between groups, even after adjusting for confounders such as smoking and seafood intake.

This study has certain limitations. First, the study results are based on a single urine sample from each participant; hence, we could not establish the reproducibility and accuracy of the measurements. A second limitation concerns control of the seafood intake. To exclude the arsenic intake from seafood, we strongly recommended participants to consume seafood for at least 2 days, but it was not strictly followed. This might have affected the interpretation of the levels of DMA. Finally, the non-exposed group should have been selected from the same community or workplace to control for environmental arsenic exposure (such as from water or air pollution), but we could not find suitable candidates among the workers’ colleagues.

This study aimed at assessing exposure to arsenic in semiconductor manufacturing workers, specifically in PM engineers of the clean process and the ion implantation process areas using urinary inorganic arsenic metabolite levels. Prior studies assessing arsenic exposure in the semiconductor industry focused mainly on ion implantation process workers, in whom the level is known to be negligible [14]. In this study, however, urinary inorganic arsenic metabolites in clean process and ion implantation PM engineers were higher than in the non-exposed group, after adjustment for smoking and seafood intake. Urinary inorganic arsenic metabolites in clean process PM engineers were the highest, suggesting that clean process engineers are exposed to arsenic. Further studies to evaluate occupational arsenic exposure in semiconductor manufacturing, especially in clean process engineers, are warranted.

## Competing interest

The authors do not have any competing interests issues in this study.

## Authors’ contributions

KWB analyzed the data and described the whole paper. YLW and HSI analyzed the metabolites of arsenics. YIH and DHK analyzed the data and participated all the discussion. EAK organized this study, analyzed the data and discussed all the results. All authors read and approved the final manuscript.
